# Correction: Histopathologic Alterations Associated with Global Gene Expression Due to Chronic Dietary TCDD Exposure in Juvenile Zebrafish

**DOI:** 10.1371/journal.pone.0106605

**Published:** 2014-08-25

**Authors:** The PLOS ONE Staff

## Notice of Republication

This article was republished on July 1st, 2014, due to the legends of Figure 6, Figure 7, and Figure 8 being ordered incorrectly. Please download the PDF again to view the corrected article. The originally published, uncorrected article and the republished, corrected article are provided here for reference.

## Supporting Information

File S1 Originally published, uncorrected article.
(PDF)

File S2 Republished, corrected article.
(PDF)
