# Supplementary material for: Histopathologic Alterations Associated with Global Gene Expression Due to Chronic Dietary TCDD Exposure in Juvenile Zebrafish
Source: PLoS One. 2014 Jul 2;9(7):e100910. doi: 10.1371/journal.pone.0100910 (PMC4079602; doi:10.1371/journal.pone.0100910)
Supplement: Table S2 — Epithelial lesions in various organs of zebrafish sampled after 28 d of dietary exposure to TCDD. (DOCX) [file pone.0100910.s002.docx]

**Table S2. Epithelial lesions in various organs of zebrafish sampled after 28 d of dietary exposure to TCDD.**

| Treatment (TCDD in diet in ppb) | Oro-pharynx^a^ | Liver^b^ | Intestine and Stomach^c^ | Exocrine Pancreas Acinar Cells^d^ | Nasal Neuro-sensory Epithelium^e^ | Fraction (%) of Ovaries with Vitellogenic Oocytes | Volume (%) of Ovaries Composed of Vitellogenic Oocytes |
| --- | --- | --- | --- | --- | --- | --- | --- |
| Control 1 | 0/10 | 0/10 | 0/10 | 0/10 | 0/3 | 6/6 (100%) | 63% |
| Control 2 | 0/10 | 0/10 | 0/10 | 0/10 | 0/3 | 5/5 (100%) | 68% |
| 1 | 0/10 | 1/10 | 0/10 | 0/10 | 0/3 | 5/5 (100%) | 70% |
| 10 | 0/10 | GD 1/10 (2+) ^f^ | 0/10 | 0/10 | 0/8 | 2/2 (100%) | 75% |
| 100 | MHP 0/10; CD 0/10 | GD 4/10 (2-3+) | 0/10 | 1/10 (2+) | UDNS, EM 5/5 (2+) | 0/5 (0%) | 0% |

**Key to Histologic Lesions**:

^a^ MHP=hyperplasia of mucous cells of distal esophagus; CD=cystic degeneration of epithelium of pharyngeal pad

^b^ GD=depletion of cytoplasmic glycogen from hepatocytes in comparison to control fish

^c^ UD=underdeveloped in comparison to control fish

^d^ DSG=depletion of secretory granules in acinar cells in comparison to control fish

^e^ UDNS, EM=neurosensory epithelium underdeveloped and lacking cilia, neuroepithelial rosette formation indicating damage to epithelium (UDNS); excess mucus filling lumina of nasal pouches

^f^  Severity of lesion: 1+=mild; 2+=moderate; 3+=severe
