# Supplementary material for: Histopathologic Alterations Associated with Global Gene Expression Due to Chronic Dietary TCDD Exposure in Juvenile Zebrafish
Source: PLoS One. 2014 Jul 2;9(7):e100910. doi: 10.1371/journal.pone.0100910 (PMC4079602; doi:10.1371/journal.pone.0100910)
Supplement: Table S3 — Epithelial lesions in various organs of zebrafish sampled after 42 d of dietary exposure to TCDD. (DOCX) [file pone.0100910.s003.docx]

**Table S3. Epithelial lesions in various organs of zebrafish sampled after 42 d of dietary exposure to TCDD.**

| Treatment (TCDD in diet in ppb) | Oro-pharynx^a^ | Liver^b^ | Intestine^c^ | Exocrine Pancreas Acinar Cells^d^ | Nasal Neuro-sensory Epithelium^e^ | Fraction (%) of Ovaries with Vitellogenic Oocytes | Volume (%) of Ovaries Composed of Vitellogenic Oocytes |
| --- | --- | --- | --- | --- | --- | --- | --- |
| Control 1 | 0/10 | 0/10 | 0/10 | 0/10 | 0/3 | 6/6 (100%) | 68% |
| Control 2 | 0/10 | 0/10 | 0/10 | 0/10 | 0/0 | 7/8 (88%) | 55% |
| 1 | 0/10 | 1/10 | 0/10 | 0/10 | 0/7 | 7/7 (100%) | 67% |
| 10 | 0/10 | GD 3/10 (2+) | 0/10 | 0/10 | 0/7 | 2/7 (29%) | 19% |
| 100 | MHP 7/10 (2+)^g^; CD^e^ 4/9 (1+); RK ^f^ 8/9 | GD 7/10 (2+) | UD 10/10 | DSG 9/9 (2+) | CD 6/6 (2+) | 0/7 (0%) | 0% |

**Key to Histologic Lesions:**

^a^ MHP=hyperplasia of mucous cells of distal esophagus; CD=cystic degeneration of epithelium of pharyngeal pad

^b^ GD=depletion of cytoplasmic glycogen from hepatocytes in comparison to control fish

^c^ UD=underdeveloped in comparison to control fish

^d^ DSG=depletion of secretory granules in acinar cells in comparison to control fish

^e^ CD=in nose, cystic degeneration of neurosensory epithelium with excess mucus filling lumina of nasal pouches; in pharyngeal pad, cystic degeneration results from clusters of dying cells forming cystic spaces in the epithelium

^f^  RK=epithelium of pharyngeal pad is less well differentiated than in controls and forms less or no keratin on its surface

^g^ Severity of lesion: 1+=mild; 2+=moderate; 3+=severe
