# Supplementary material for: Histopathologic Alterations Associated with Global Gene Expression Due to Chronic Dietary TCDD Exposure in Juvenile Zebrafish
Source: PLoS One. 2014 Jul 2;9(7):e100910. doi: 10.1371/journal.pone.0100910 (PMC4079602; doi:10.1371/journal.pone.0100910)
Supplement: Table S4 — Mesenchymal lesions in various organs of zebrafish sampled after 28 d of dietary exposure to TCDD. (DOCX) [file pone.0100910.s004.docx]

**Table S4. Mesenchymal lesions in various organs of zebrafish sampled after 28 d of dietary exposure to TCDD.**

| Treatment (TCDD in diet in ppb) | Reduced Hemopoietic Cells in Kidney Marrow | Anemia Evident in Peripheral Blood | Foreshortened Maxilla | Reduced Abdominal Adipose Tissue |
| --- | --- | --- | --- | --- |
| Control 1 | 0/10 | 0/10 | 0/10 | 0/10 |
| Control 2 | 0/10 | 0/10 | 0/10 | 0/10 |
| 1 | 0/10 | 0/10 | 0/10 | 0/10 |
| 10 | 0/10 | 0/10 | 0/10 | 0/10 |
| 100 | 0/10 | 6/10 | 0/10 | 4/10 |
