# Supplementary material for: Histopathologic Alterations Associated with Global Gene Expression Due to Chronic Dietary TCDD Exposure in Juvenile Zebrafish
Source: PLoS One. 2014 Jul 2;9(7):e100910. doi: 10.1371/journal.pone.0100910 (PMC4079602; doi:10.1371/journal.pone.0100910)
Supplement: Table S6 — Edema syndrome lesions in zebrafish sampled after 28 d weeks of dietary exposure to TCDD. (DOCX) [file pone.0100910.s006.docx]

**Table S6. Edema syndrome lesions in zebrafish sampled after 28 d weeks of dietary exposure to TCDD.**

| Treatment (TCDD in diet in ppb) | Pericardial Edema | Ascites | Edema in Ovarian Stroma | Retrobulbar edema |
| --- | --- | --- | --- | --- |
| Control 1 | 0/8 | 0/10 | 0/6 | 0/10 |
| Control 2 | 0/9 | 0/10 | 0/5 | 0/10 |
| 1 | 0/9 | 0/10 | 0/5 | 0/10 |
| 10 | 0/7 | 0/10 | 0/2 | 0/10 |
| 100 | 7/10 (1-2+) ^a^ | 0/10 | 5/5 (2-3+) | 3/10 (2+) |

^a^ Severity of lesion: 1+=mild; 2+=moderate; 3+=severe
