# Supplementary material for: Correction: Histopathologic Alterations Associated with Global Gene Expression Due to Chronic Dietary TCDD Exposure in Juvenile Zebrafish
Source: PLoS One. 2014 Aug 25;9(8):e106605. doi: 10.1371/journal.pone.0106605 (PMC4143344; doi:10.1371/journal.pone.0106605)
Supplement: File S2 — Republished, corrected article. (PDF) [file pone.0106605.s002.pdf]

Histopathologic Alterations Associated with Global Gene Expression Due to Chronic Dietary TCDD Exposure in Juvenile Zebrafish

Qing Liu[1,2], Jan M. Spitsbergen[3], Ronan Cariou[4], Chun-Yuan Huang[5], Nan Jiang[6], Giles Goetz[7], Reinhold J. Hutz[1], Peter J. Tonellato[5,8,9], Michael J. Carvan III[1,2]*

1 Department of Biological Sciences, University of Wisconsin-Milwaukee, Lapham Hall, Milwaukee, Wisconsin, United States of America, 2 School of Freshwater Sciences, University of Wisconsin-Milwaukee, Milwaukee, Wisconsin, United States of America, 3 Department of Microbiology, Oregon State University, Nash Hall, Corvallis, Oregon, United States of America, 4 LUNAM Université, Oniris, Laboratoire d'Etude des Résidus et Contaminants dans les Aliments (LABERCA), Nantes, France, 5 Zilber School of Public Health, University of Wisconsin-Milwaukee, Milwaukee, Wisconsin, United States of America, 6 Roche NimbleGen, Inc., Madison, Wisconsin, United States of America, 7 School of Aquatic and Fishery Sciences, University of Washington, Seattle Washington, United States of America, 8 Department of Pathology, Beth Israel Deaconess Medical Center, Boston, Massachusetts, United States of America, 9 Center for Biomedical Informatics, Harvard Medical School, Boston, Massachusetts, United States of America

Abstract

The goal of this project was to investigate the effects and possible developmental disease implication of chronic dietary TCDD exposure on global gene expression anchored to histopathologic analysis in juvenile zebrafish by functional genomic, histopathologic and analytic chemistry methods. Specifically, juvenile zebrafish were fed Biodiet starter with TCDD added at 0, 0.1, 1, 10 and 100 ppb, and fish were sampled following 0, 7, 14, 28 and 42 d after initiation of the exposure. TCDD accumulated in a dose- and time-dependent manner and 100 ppb TCDD caused TCDD accumulation in female (15.49 ppb) and male (18.04 ppb) fish at 28 d post exposure. Dietary TCDD caused multiple lesions in liver, kidney, intestine and ovary of zebrafish and functional dysregulation such as depletion of glycogen in liver, retrobulbar edema, degeneration of nasal neurosensory epithelium, underdevelopment of intestine, and diminution in the fraction of ovarian follicles containing vitellogenic oocytes. Importantly, lesions in nasal epithelium and evidence of endocrine disruption based on alternatively spliced *vasa* transcripts are two novel and significant results of this study. Microarray gene expression analysis comparing vehicle control to dietary TCDD revealed dysregulated genes involved in pathways associated with cardiac necrosis/cell death, cardiac fibrosis, renal necrosis/cell death and liver necrosis/cell death. These baseline toxicological effects provide evidence for the potential mechanisms of developmental dysfunctions induced by TCDD and *vasa* as a biomarker for ovarian developmental disruption.

Citation: Liu Q, Spitsbergen JM, Cariou R, Huang C-Y, Jiang N, et al. (2014) Histopathologic Alterations Associated with Global Gene Expression Due to Chronic Dietary TCDD Exposure in Juvenile Zebrafish. PLoS ONE 9(7): e100910. doi:10.1371/journal.pone.0100910

Editor: Robert L. Tanguay, Oregon State University, United States of America

Received February 7, 2014; **Accepted** May 31, 2014; **Published** July 2, 2014

Copyright: © 2014 Liu et al. This is an open-access article distributed under the terms of the Creative Commons Attribution License, which permits unrestricted use, distribution, and reproduction in any medium, provided the original author and source are credited.

Funding: This work was funded by the University of Wisconsin Sea Grant Institute under grants from the National Sea Grant College Program, National Oceanic and Atmospheric Administration, U.S. Department of Commerce, and from the State of Wisconsin. Federal grant number NA060AR4170011, project number R/BT-21. This work was also supported by the UWM Children's Environmental Health Sciences Core Center (NIEHS 2P30ES004184), and the Shaw Fund of the Greater Milwaukee Foundation. The funders had no role in study design, data collection and analysis, decision to publish, or preparation of the manuscript.

Competing Interests: Nan Jiang is employed by Roche NimbleGen. His contribution to this study is not related to his employment. All other authors declare that they have no conflict of interest. This does not alter the authors' adherence to PLOS ONE policies on sharing data and materials.

* Email: carvanmj@uwm.edu

Introduction

TCDD is a highly toxic, widespread environmental chemical that is a by-product of incinerating plastics, chlorinated industrial chemicals and other hydrocarbons. TCDD has been shown to persist in the environment, bioaccumulate in humans and wildlife through trophic transfer [1] and contributes to adverse disease-specific effects such as cancer and cardiovascular toxicity [2–5]. Zebrafish, as an aquatic animal model, continues to play a central role in TCDD toxicological developmental and genetic research [6]. TCDD has been found to cause precursor and disease-associated adverse effects in zebrafish embryos, such as craniofacial malformations in jaw, edema in yolk sac and pericardium, decreases in cardiac output [7], spinal deformity [8], disruption of development of common cardinal vein [9], and a developmental reduction in blood flow in mesencephalic vein [10]. In TCDD-treated adult zebrafish, collections of fully developed disease phenotypes and dysfunction have been documented. For example, TCDD has been found to inhibit caudal fin regeneration in zebrafish, suggesting dysfunction of tissue's self-repairing abilities by its inhibition of cellular differentiation and proliferation [11]. In the reproductive system, TCDD represses the follicle maturation in ovarian development and inhibits estradiol biosynthesis [12].

In spite of these wide-ranging and impactful early embryonic dysregulation findings and fully developed adult disease implications, there are few studies examining the onset and development of the dysregulation in fish during the critical developmental transition from embryo to adult. For example, neither toxicological nor toxicogenomic effects of dietary TCDD exposure on juvenile zebrafish are well documented. Although it is well

understood that key dysregulation critical to adult health are initiated in the embryonic stage, many diseases and exposure-pathology emerge during the juvenile stages (e.g. so called "early" onset diseases) or precursors to the adult problems are not observed in embryonic studies may be identified in the juvenile stage. There is a gap in our understanding of the pathology, gene expression and dysregulation of juvenile zebrafish due to dietary TCDD exposure. We hypothesize that early onset precursors to adult diseases and toxicogenomic dysregulation can be detected in juvenile fish and that juveniles may exhibit dysregulation not observed in either embryos or adults.

The objective of this study is to create a baseline of evidence of the phenotypes and genetic dysregulation leading to a better understanding of the molecular mechanism (s) involved in the onset of TCDD-induced disease exhibited in zebrafish. Chronic dietary TCDD exposure was conducted by exposing juvenile zebrafish to dietary TCDD at 0, 0.1, 1, 10 or 100 ppb TCDD for 42 d. Accumulations of TCDD in fish were measured in whole fish bodies. TCDD-induced toxicities were investigated using histopathologic analysis, and global gene expression in fish treated with TCDD was analyzed through microarray experiments. TCDD accumulation was dose- and time-dependent in zebrafish, although no significant mortality was observed even at accumulated doses up to 10 ppb. Juvenile zebrafish showed pathologic alterations due to TCDD exposure at 28 and 42 d, especially in disruption of ovarian development and degeneration of nasal epithelium. Functional analysis of microarray data from TCDD-treated fish revealed a correlation between gene expression and histopathologic outcomes.

Methods

Ethics statement

Zebrafish were handled following protocols approved by the Animal Care and Use Committee (ACUC) of the University of Wisconsin–Milwaukee (UWM).

Chemicals and diet

TCDD was purchased from the National Cancer Institute–Chemical Carcinogen Reference Standard Repository, Midwest Research Institute (Kansas City, MO) and stored at room temperature. Dose formulations for treatment were prepared by dissolving TCDD in ethanol and mixing the appropriate amount into Biodiet starter #2 (Bio-Oregon, Inc., Warrenton, OR), using 1 L ethanol per kilogram of diet. The ethanol in the mixture was evaporated under a fume hood to remove the vehicle.

Animals

Juvenile wild-type zebrafish (stock originally purchased from EkkWill Waterlife Resources, Ruskin, FL) (0.16 g, 2.10 cm) were reared in 28°C dechlorinated, filtered municipal water (DFMW) in the NIEHS Children's Environmental Health Sciences Core Center (CEHSC) animal facility. Fish from a single treatment were housed in a single flow-through 40 L polycarbonate tank to reduce hazardous waste. Zebrafish were fed Biodiet starter (4% body weight per day) for 42 d with TCDD added at 0 ppb, 0.1 ppb, 1 ppb, 10 ppb or 100 ppb (nominal concentration, ethanol used as vehicle). Each treatment had one tank with 300 fish (N = 300). Throughout any animal treatment, fish were visually monitored three to four times daily as part of normal husbandry. Any individual that exhibited signs of distress (the inability to maintain an upright position, holding fins close to body for a prolonged period, erratic swimming, remaining close to the bottom or top of the tank, inflammation around the gills, excessive and/or difficult respiration, or suppression of pigmentation) was immediately humanely euthanized. For euthanasia, fish were anaesthetized in 100 mg/l of buffered MS222 (tricaine mesylate) solution, then, killed by cervical transection or immersion in liquid nitrogen. Fifty fish were collected at 7, 14, 28 or 42 days (d) respectively, following initiation of TCDD diet, and 15 of them were measured for body weight and length. Samples were stored at −80°C until used for experiments.

TCDD assessment

All samples have been analyzed by the French National Reference Laboratory for PCDD/Fs and PCBs in food and feed, using the method described below that was accredited according to the ISO 17025 standard. Details of the sample preparation method can be found elsewhere [13–15].

Five individuals of each group were pooled for extraction using a Pressurized Liquid Extraction system (ASE, Dionex, Sunnyvale, CA, USA). Three successive static extraction cycles (5 min each) were performed using a mixture of toluene/acetone (70:30, v/v) at 100 bar and 120°C. Extracts were aliquoted before purification steps, according to expected TCDD content. Clean-up and fractionation of TCDD was carried out using liquid chromatography columns with sulphuric acid silica, Florisil and carbon (Carbopack C) in that order.

All samples were analyzed by gas chromatography coupled to high resolution mass spectrometry (GC-HRMS). Identification and quantification of TCDD was achieved using the isotope-dilution method. $^{13}C_{12}$-labeled TCDD was used as internal standard. GC separation was performed on a DB-5MS capillary column (30 m×0.25 mm, 0.25 μm) in HP 7890 instrument (HP, Palo Alto, CA, USA). HRMS measurements were achieved on a JMS 800D electromagnetic instruments (Jeol, Tokyo, Japan), operating at a resolution of 10,000 in the selected ion-monitoring acquisition mode after Electroionisation (42 eV).

Histopathologic analysis

Five to ten fish were collected after 28 and 42 d of dietary exposure from each treatment group for histopathologic analysis. Briefly, fish were anaesthetized in 100 mg/l of MS222, and then sacrificed by cervical transection. The coelomic cavity was opened allowing perfusion of internal organs. A transverse incision at the level of the anterior spinal cord enhanced fixation of brain and kidney. Tails were removed, and carcass was placed into plastic cassettes separately. The samples were fixed in Davison's solution (150 ml ethanol, 95%; 100 ml formaldehyde 37%, 50 ml glacial acetic acid and 58 ml distilled water) for 24 h, rinsed in cold tap water (24 h), decalcified in Cal-EX for 24 h (Fisher Scientific, Pittsburgh, PA), and rinsed again in cold tap water (24 h). Dehydration occurred in 50% and then 70% ethanol for 24 h each. The samples were subsequently embedded in paraffin. Sagittal-step sections were cut near midline and medial to the eye (2–4 per fish), mounted on glass slides and stained with hematoxylin and eosin by Mass Histology Service, Inc. (Worcester, MA). Histopathologic analysis was performed at Oregon State University (Corvallis, OR) by a veterinary pathologist certified by the American College of Veterinary Pathologists, having 30 years of experience in fish pathology research and diagnostic pathology.

RNA isolation

Total RNA from individual fish was isolated using Trizol reagent (Invitrogen, Carlsbad, CA). One ml of Trizol was used per individual fish, which were then homogenized with Power Gen 500 (Fisher Scientific, PA). Chloroform (0.2 ml) was added for dissociation of nucleoprotein and mixed by shaking the tubes by

hand for 15 s. The samples were then centrifuged at 12000 g for 15 min at 8°C. The upper aqueous phase was moved to a clean 1.5 ml microcentrifuge tube, and 0.5 ml of isopropyl alcohol was added for precipitation of the RNA by incubating the samples at room temperature for 10 min. The samples were centrifuged at 12000 g for 15 min at 8°C. Then the supernatant was removed and 1 ml of 75% ethanol was added to wash the pellet, and the samples were centrifuged at 12000 g for 5 min at 8°C. The samples were air-dried and then dissolved in RNase-free water. The concentration of RNA was determined with a ND-1000 NanoDrop Spectrophotometer (Thermo Scientific, DE).

RNA purification was performed using the RNeasy MiniElute cleanup kit (Qiagen, CA) following the manufacturer's protocol. Pre-cleaned total RNA samples (<45 µg in a total volume of 100 µl RNase-free water) were digested with 2.5 µl DNase I (1500 U) for 10 min at room temperature. Digested RNA was eluted through a RNeasy MinElute Spin Column with 350 µl RLT buffer and 250 µl 100% ethanol to bind RNA to the membrane of the column. The column was washed with 500 µl 80% ethanol after being washed with 500 µl RPE buffer, and RNA was eluted in 20 µl RNase-free water. The concentration of RNA was determined using the ND-1000 NanoDrop Spectrophotometer. Quality of zebrafish total RNA was determined with an Experion RNA StdSens chip (Bio-Rad, CA).

Sex designation

Zebrafish at the juvenile stage were placed into the TCDD feeding experiment and typically achieve gonadal maturation after 3–4 weeks. The body cavity of each fish was opened and the presumptive sex determined by visual assessment of the gonad. Unexpectedly, all fish in the 100 ppb-treated group were lacking mature or undifferentiated gonads at 42 d.

We felt the need to determine the sex of each individual fish to account for differences in response between males and females. Since visual sex-determination is not 100% reliable, a novel RT-PCR-based sexing method was developed and validated. It has been reported that male and female zebrafish have two different splicing variants of the gene *vasa* after 25 dpf that differ in length depending on inclusion or exclusion of exon 4 [16]. Male zebrafish mainly express short *vasa* mRNA without exon 4 and have very low expression of long *vasa* containing exon 4, and female zebrafish have both short and long *vasa* mRNA splice variants (Figure 1). In order to distinguish male and female by RT-PCR based on the numbers of bands of RT-PCR products on the gel, a pair of primers was designed across from exon 2 to exon 6 as shown in Figure 1. Both visual observation of the gonad and PCR with vasa primers were used to sex TCDD-treated zebrafish (Table 1).

Quantification of exon-4 transcripts of *vasa* mRNA

In order to identify presumptive TCDD-induced disruption of splicing of vasa mRNA between male and female zebrafish, another pair of primers was designed for testing levels of exon-4 transcripts in two qRT-PCR experiments: 1) sexed male and female zebrafish in all treatment groups at 28 d; and 2) sexed male, female and immature zebrafish in 0 and 100 ppb groups at each collection time point.

For quantification of the exon-4 transcripts in qRT-PCR experiments, RNA from female and male fish in 0 ppb groups at 28 d was pooled and used as a common reference for the comparison. We identified guanine nucleotide-binding protein (G protein), beta polypeptide 2-like 1 (*gnb2l1*) as a normalizer gene based on this and previous studies of TCDD-treated zebrafish in this laboratory. The sequences of primers for *gnb2l1* are: gnb2l1_forward: 5′-AGCTGAGGCAGGACATCATT-3′; gnb2l1_reverse: 5′-GCTTTATCTGGTTCCGATGG-3′.

Regarding QPCR, first-strand cDNA was synthesized from 1 µg of total RNA from individual fish with oligo-dT using an AffinityScriptTM Multi Temperature cDNA synthesis Kit (Agilent Technologies, Inc. Santa Clara, CA) following the manufacturer's protocol. PCR amplification was performed with the Mx3000P QPCR Systems (Agilent) in 10 µl reactions using 1 µl of cDNA (10 ng of total input RNA), 200 nM each of forward and reverse primer and 1×Power SYBR Green PCR Master Mix (Applied Biosystems, Carlsbad, California). The real-time PCR program consisted of 1 cycle of 95°C for 9 min; and 40 cycles of 95°C for 15 s, 59°C 30 s and 72°C for 30 s, with fluorescence detection at the end of each 59°C step. The products were determined by melting curves. Relative gene expression data (fold-changes) were calculated using the mathematical model described by Pfaffl [17]. Statistical analysis of QPCR data was performed using SigmaStat 11.0 (Systat Software, Inc., CA). One-way analysis of variance (ANOVA) was used to detect the significances of treatments compared to control group at 28 d.

Microarray experiments

We used individual fish to perform the microarray experiments and assess biologic variability in gene expression. Two microarray experiments were designed based on time, dose, and designated sex (Figure 2): (1) Dose-dependent experiment: gene expression testing conducted in TCDD-treated (at 0, 0.1, 1, 10 or 100 ppb) for male and female sexed zebrafish at 28 d; and (2) Time-dependent experiment: gene expression testing of 100 ppb-TCDD treated male and female sexed zebrafish at 7, 14, 28 and 42 d.

NimbleGen Gene Expression 12×135 K zebrafish microarrays were used for genome-wide expression analysis of TCDD-treated zebrafish. RNA samples from six zebrafish (three male and three

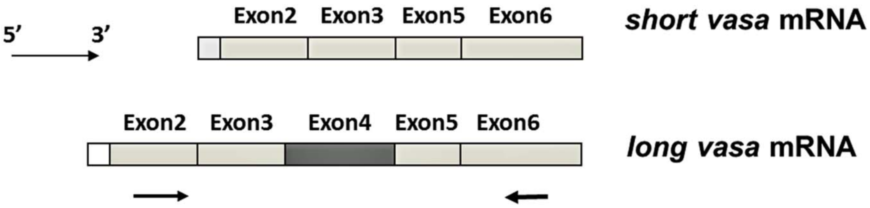

Figure 1. Two *vasa* splicing variants in zebrafish (Krøvel *et al.*, 2004). Male zebrafish have short vasa mRNA and female zebrafish have both short and long vasa mRNA. A pair of primers was designed crossing exon 2 and exon 6 (arrow). The sequences of primers are: vasa_long_forward 5′-CCCAATATGGATGACTGGGAG-3′; vasa_long_reverse 5′-CAGCACCTCCTGTATAAAAGC-3′. A PCR method was applied to distinguish male and female based on the numbers of bands of PCR products on the gel. Female fish have two bands and male fish have one band. In addition, expression of exon 4 within vasa gene was quantified by real-time QPCR. The sequences are: exon4_forward: 5′-CTTCACGCGTGTCCACCTG-3′; exon4_reverse: 5′-GCTCTTGAATCCTCCATCAGAACC-3′.
doi:10.1371/journal.pone.0100910.g001

Table 1. Sex designation of dietary TCDD-treated zebrafish using both observation and QPCR method.

Visual observation	PCR by vasa	Designated sex
testis	male	male
ovary	female	female
Abnormal gonad	female	female (immature)

doi:10.1371/journal.pone.0100910.t001

female, when available) were randomly selected. cDNA was synthesized using the Superscript Double-Stranded cDNA synthesis Kit (Invitrogen, Carlsbad, CA). Ten µg total RNA from individual fish was denatured at 70°C for 5 min with oligo-dT primers, and then was reverse transcribed to the first stand cDNA at 42°C for 60 min with SuperScript II enzyme. The second-strand cDNA was synthesized at 16°C with DNA ligase, DNA polymerase I and RNaseH for two hours. After digestion with RNase A solution, the double-stranded DNA was cleaned in a Phase Lock tube (Fisher Scientific, PA) with phenol: chloroform: isoamyl alcohol (25:24:1, Ambion, NY), and then precipitated in ice-cold ethanol with 7.5 M ammonium acetate and 5 mg/ml glycogen, and dried to a pellet in a DNA 120 SpeedVac (Thermo Scientific, DE). The DNA was then cleaned using QIAquick PCR purification kit (Qiagen, CA). The quality of DNA was determined with an Experion RNA HighSens chip (Bio-Rad, CA). One µg double-stranded DNA was used for Cy3-labeling reaction with random primer and Klenow fragment (3′–>5′ exo-) using a One-Color DNA labeling Kit (NimbleGen, WI). Cy3-labeled DNA was precipitated in ice-cold ethanol with 5 M NaCl and dried to a pellet in a SpeedVac, then rehydrated in nuclease-free water. The concentration of Cy3-labeled DNA was determined using a ND-1000 NanoDrop Spectrophotometer. Cy3-labeled double stranded DNA was hybridized to a NimbleGen 12×135 K array using a NimbleGen hybridization kit; the hybridization was performed in the NimbleGen Hybridization System (NimbleGen, WI) at 42°C for 16 h. Arrays were washed with NimbleGen Wash buffers and 1 M DTT, and then spin dried in a NimbleGen microarray drier.

Hybridized microarrays were sent to MOgene, LC (St, Louis, MO) for scanning. The arrays were scanned at 2 µm resolution on a NimbleGen MS200 scanner with auto-gain adjust. The TIFF images were gridded and signals extracted using NimbleScan v. 2.6. Expression data were normalized with the Robust Multichip Average (RMA) algorithm as described previously [18].

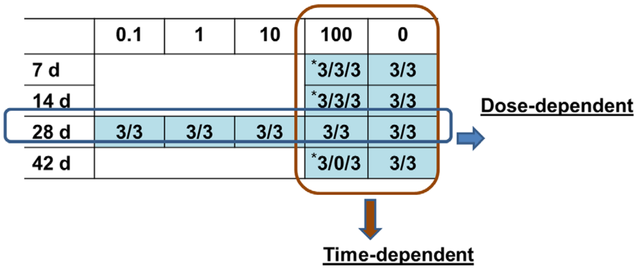

Figure 2. Microarray experiment design for TCDD-treated zebrafish. Zebrafish were fed with TCDD at 0, 0.1, 1, 10 and 100 ppb TCDD in food for 42 d. Fish were collected at 7, 14, 28 and 42 d. Two microarray experiments were conducted. Dose-dependent experiment: fish from each treatment at 28 d for microarray analysis; Time-dependent experiment: fish from 0 and 100 ppb groups at 7, 14, 28 and 42 d for microarray experiment. Three female and three male fish from each group were used for microarray experiments with NimbleGen 12×135 K zebrafish microarray system was used for this project. The asterisk (*) means three immature fish in 100 ppb groups at 7, 14 and 42 d were used for microarrays, and no mature female fish were found in 100 ppb group at 42 d.
doi:10.1371/journal.pone.0100910.g002

Microarray data analysis

Differential expression analysis was performed with Multiple Array Viewer (MeV, Dana-Farber Cancer Institute, MA) software, version 4.8.1 [19,20]. Three statistical methods were reviewed and tested for analysis: Bayesian Estimation of Temporal Regulation (BETR), one-way ANOVA, and Significance Analysis of Microarrays (SAM). False discovery rate (FDR) was controlled with the Benjamini and Hochberg procedure [21]. For both experiments and all expression data, BETR consistently predicted the lowest FDR of the three methods, and was therefore selected for differential expression analysis. Statistical significance was set at FDR <0.05. We applied K-means clustering using MeV software to cluster genes based on their expression profiles across doses and time.

Gene Ontology (GO) annotations (categories of biological process [BP]) were achieved by performing BLASTX between array sequences against all protein sequences of the Amigo database at amigo.geneontology.org [22]. Zebrafish sequences were extracted to align against the Amigo database based on their accession numbers on the array from different sources, including the NCBI (http://www.ncbi.nlm.nih.gov/UniGene/), VEGA (http://vega.sanger.ac.uk/Danio_rerio/) and Ensembl (http://www.ensembl.org/Danio_rerio). The "GOHyperGAll" script from BioConductor was employed to conduct GO enrichment analysis for functional annotations of dysregulated genes. Enriched GO terms are statistically significant and representative in dysreuglated genes against the whole array. Ingenuity Pathways Analysis (IPA) software (Ingenuity Systems, www.ingenuity.com) was used to analyze IPA Canonical pathways and generate IPA Tox lists. Canonical pathways analysis identified the most significant pathways from the IPA library from the input data set. Tox lists were most significant toxicity lists identified from the IPA library [23], and were associated with key toxicological responses to xenobiotic insult. The p values were calculated by Fisher's exact test, which calculates the probability of a gene in the dataset of a given size due to chance alone. Since IPA software does not support zebrafish data, we used human orthologs of dysregulated genes from NimbleGen geneID file for knowledge-based pathway and functional analysis. Microarray data (both pre- and post-normalized) have been deposited in the Gene Expression Omnibus (GEO) database (http://www.ncbi.nlm.nih.gov/geo/). The data is accessible through GEO Series accession number GSE47434 for the dose-dependent experiment, and GSE47547 for the time-dependent experiment.

Results

Mortality, growth and bioaccumulation of zebrafish treated with TCDD

No significant mortality was observed in any TCDD treatment group after 42 d of feeding (data not shown) by Kaplan-Meier survival analysis with log-rank significance test ($p<0.05$). 10 ppb- and 100 ppb TCDD caused 10% and 23% mortality in treated zebrafish at 42 d, respectively. Body weight and length and condition factor of TCDD-treated are shown in Table S1. Significant decreases of body weight and length were seen in 100 ppb-treated male zebrafish at 28 d. Significant decreases of body length were observed in male zebrafish in the 10 and 100 ppb TCDD-treatment groups at 28 d. Condition factor of female zebrafish was significantly decreased by 100 ppb at 28.

During 42 d of feeding, accumulation of TCDD in whole body exhibited dose- and time-dependent increases in both female and male fish (Figure 3). Due to the detection sensitivity of the instrument, we had to pool fish samples from each group for a single TCDD measurement, thus there were no technical replicates. The amount of TCDD in female fish in the 100-ppb group achieved 15.49 ppb at 28 d. In male fish, the amount of TCDD in male fish in 100 ppb group achieved highest concentrations (18.04 ppb) at 28 d, but it decreased to 5.33 ppb at 42 d. In the 1 ppb group, there was a technical failure during analysis and the samples were destroyed, therefore, we lack TCDD assimilation data of in the 1-ppb group at 14 d. In addition, no fish were identified as female by physical observation at 42 d.

TCDD-induced clinical toxicity and gross and microscopic lesions in zebrafish

After 28 days of exposure to TCDD, some zebrafish (less than 10%) in the 100 ppb group showed hemorrhaging around their gills. These effects became more severe at 42 days. Histopathologic results were characterized as three categories: epithelial lesions, mesenchymal lesions and edema syndrome. Male and female zebrafish exhibited similar degrees of lesions, which were mainly observed in the 10 and 100 ppb TCDD-treatment groups. Thus, we combined histopathologic data from both males and females, and the summary of lesions is shown in Figure 4. Between 28 and 42 days, the TCDD-induced pathologic alterations exhibited time- and dose-dependencies in various organs of TCDD-treated zebrafish.

Detailed information of epithelial lesions is shown in Table S2 (28 d) and Table S3 (42 d). Briefly, epithelial lesions were observed in oropharynx (hyperplasia of esophagus and degeneration of pharyngeal pad), liver (depletion of glycogen), intestine (underdevelopment), pancreas (depletion of secretory granules in acinar cells), nose (underdevelopment and degeneration of neurosensory epithelium, Figure 5), and ovary (decreases in the fractions of vitellogenic oocytes compared to control fish, Figure 6). The evidence of TCDD-induced lesions in nasal epithelium of fish is novel. TCDD-induced degeneration of nasal epithelium in zebrafish is rarely documented. In this project, 100 ppb TCDD-treated zebrafish showed highly disorganized and poorly differentiated hyperplastic neurosensory epithelium compared to well differentiated, orderly, and uniform neurosensory epithelium in control fish (Figure 5).

TCDD-induced lesions in mesenchymal tissues (Tables S4 and S5) included decreases in hemopoietic cells in kidney marrow (Figure 7), anemia in peripheral blood, foreshortened maxilla and diminution in abdominal adipose tissue. Edema syndromes (Tables S6 and S7) were observed in pericardium (Figure 8), posterior regions of the eyeball, and in stroma of the ovary (Figure 6).

After 28 d of dietary exposure, a small percentage (less than 20%) of 100 ppb-treated zebrafish were visually determined to be female by physical observation. At 42 d, none of the fish showing in the 100 ppb group could be determined as female by physical observation of the gonad. In contrast, female zebrafish in the control and low TCDD-treatment groups possessed matured ovaries, and overall, there was an equal number of males and females. Perinucleolar and cortical alveolar stages are two of the oocyte developmental stages that occur prior to the vitellogenic stage. In the perinucleolar stage, follicular cells are monolayer and oocytes are large. In cortical alveolar stages, yolk vesicles (cortical

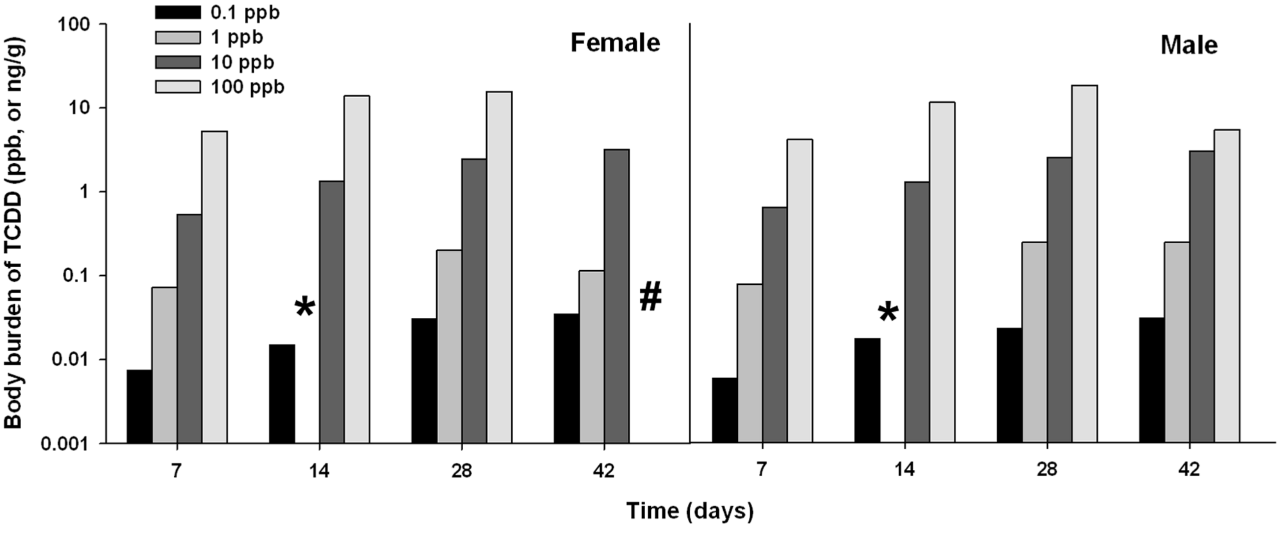

Figure 3. Assimilation of TCDD in female and male zebrafish during 42-d of dietary exposure. TCDD from extraction of pooled fish (up to 5) were analyzed by gas chromatography coupled to high-resolution mass spectrometry (GC-HRMS). The data (ppb) represent the observed TCDD (ng) verse wet weight of fish (g). The asterisk (*) means failed analysis and no more samples to be detected in fish treated with 1 ppb TCDD at 14 d; the "#" means no fish in 100 ppb group were identified as female by physical observation at 42 d.
doi:10.1371/journal.pone.0100910.g003

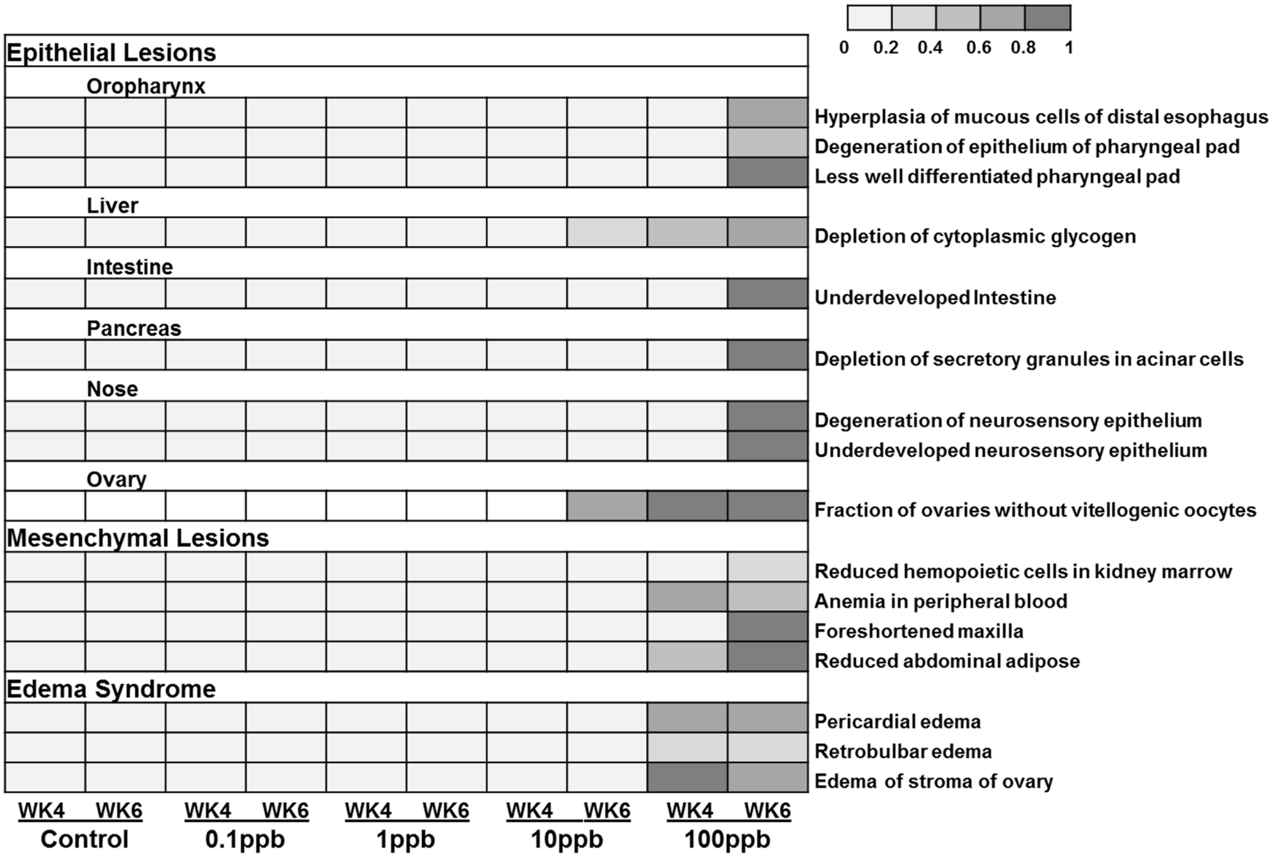

Figure 4. Heat-map of lesions found in dietary TCDD-treated zebrafish. Fish were collected at 28 d and 42 d after starting of dietary TCDD exposure. Lesions were characterized as three main categories: epithelial lesions, mesenchymal lesions and edema syndrome. Each square represents the percentage of fish that had the lesions.
doi:10.1371/journal.pone.0100910.g004

alveoli) increase in size and can be observed in the follicle. Based on the histopathologic outcomes in Figure 6, perinucleolar and cortical alveolar oocytes accounted for a larger percentage in the ovary of the 100 ppb TCDD-treated zebrafish than in control, indicating that the development of the ovary in zebrafish was disrupted by chronic dietary TCDD exposure. Combining this information with sex identification by vasa expression in Table 1 (which shows that many fish had female vasa-expression features even though no mature gonads were observed) further supports our view that development of the ovary was disrupted by exposure.

Gene expression analysis in TCDD-treated zebrafish

Dose-dependent experiment (0.1, 1, 10 and 100 ppb TCDD at 28 d). One hundred twenty-nine and 2106 genes were found to be differentially expressed in female and male zebrafish treated with TCDD at 28 d, respectively (Tables S8 and S9). Only 38 genes were shared in common between female and male zebrafish.

Clustering analysis of gene expression patterns and top five enriched ($p<0.05$) GO terms (biological process category) for each cluster are shown in Figure 9. In female zebrafish, cluster 1 (44

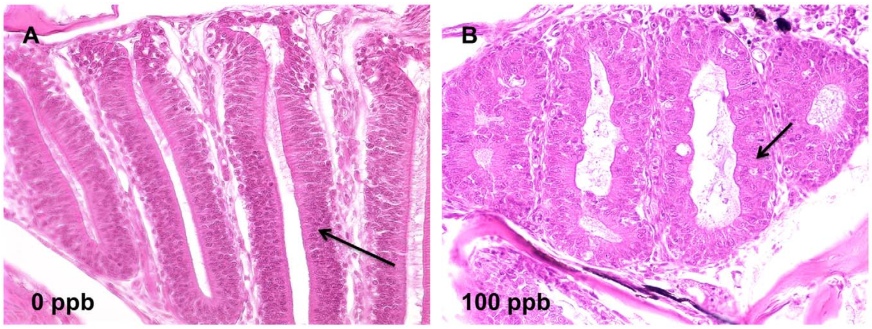

Figure 5. TCDD caused cystic degeneration in nasal epithelium in zebrafish after 42 d of dietary exposure. A) Well differentiated, orderly, uniform lamella (arrow) of zebrafish in 0 ppb group; B) cystic degeneration of nasal epithelium (arrows) was found in 100 ppb TCDD-treated zebrafish at 42 d (40X).
doi:10.1371/journal.pone.0100910.g005

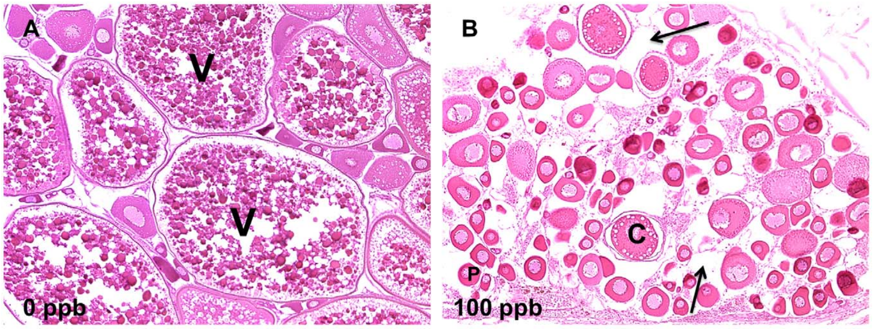

Figure 6. TCDD caused disruption of ovary development in zebrafish at 42 d. A) Ovulated vitellogenic follicles (V) in mature ovary of zebrafish in 0 ppb group; B) immature ovary in fish treated with 100 ppb TCDD. Ovary lacks vitellogenic follicles. Moderate interstitial edema (arrows) was observed; only previtellogenic oocytes are present: cortical alveolar oocytes (C) and perinucleolar oocytes (P) (10X).
doi:10.1371/journal.pone.0100910.g006

genes) showed genes primarily down-regulated by TCDD at different concentrations, and these genes are mainly involved in lipid transport and lipoprotein assembly; cluster 2 (33 genes) shows genes primarily up-regulated by TCDD at different concentrations, and these are mainly involved in membrane organization and G-protein signaling pathways; genes in cluster 3 (52 genes) were up-regulated by TCDD at 0.1 and 1 ppb but down-regulated by 100 ppb-TCDD, and these genes are shown to be involved in negative regulation of DNA replication and metabolic process.

Dysregulated genes in TCDD-treated male zebrafish were divided into five clusters. Genes in cluster 1 (404 genes) were down-regulated in all groups except in the 10 ppb group; GO terms such as proteolysis, endothelial cell proliferation, steroid hormone receptor signaling pathway, cell proliferation and serotonin transport were overrepresented in these genes. Genes in cluster 2 (391 genes) were down-regulated in the 0.1 and 1 ppb groups but up-regulated in the 100 ppb group; and these are mainly involved in visual perception of light stimulus and sensory perception. Genes in cluster 3 (581 genes) were primarily up-regulated by 100 ppb of TCDD, and their enriched GO terms are related to brain development and central nervous system development. Cluster 4 contains 469 genes that were up-regulated in the 0.1, 1 and 10 ppb groups but down-regulated in the 100 ppb group, with the enriched GO terms being myofibril assembly, cellular component assembly involved in morphogenesis, actomyosin structure organization, striated muscle cell development and muscle cell development. Cluster 5 contains 262 genes that were up-regulated at each concentration of TCDD, and these genes are primarily involved in cellular response to stimulus. These dysregulated genes were analyzed by IPA software and groups of genes were mapped to IPA Canonical pathways and Tox Lists (Table S11). A series of IPA Tox lists was identified between female and male fish, such as renal necrosis/cell death, NRF2-mediated oxidative stress response, and cell cycle regulation; and common significant Canonical pathways were cardiac hypertrophy signaling, glycolysis/gluconeogenesis, p53 signaling. The representative top Canonical pathways and Tox lists are shown in Table 2.

Time-dependent experiment (100 ppb TCDD at 7, 14 d or 42 d). Statistically expressed genes in 100 ppb TCDD groups at 7, 14 and 42 days are shown in Table S10. Thirteen, 128 and 77 genes were differentially expressed in female, immature female and male zebrafish, respectively, and were identified at 7 and 14 d after initiation of 100 ppb TCDD exposure; none of these were shared in common among groups (Figure 10A). Two hundred seventy-eight, 2833 and 1029 genes were found to be significantly dysregulated by 100 ppb TCDD at 14 d in the respective groups above, and only three genes were common dysregulated genes (Figure 10B). At 42 d, 623 and 4604 genes were significantly dysregulated in immature female zebrafish and male zebrafish, respectively; and 98 genes were common dysregulated genes between immature female and male zebrafish (Figure 10C). We mapped the differentially expressed genes to Canonical Pathways and Tox Lists with IPA, and statistically significant ($p<0.05$) pathways and lists are shown in Table S12. Based on the Tox Lists, liver necrosis/cell death, renal necrosis/cell death and TGF-β signaling were assigned as dysregulated genes in TCDD-treated fish.

Based upon physical observation and histopathologic analysis, none of the female fish treated with 100 ppb showed matured ovaries, and fraction (%) of ovaries with vitellogenic oocytes was near zero (Figure 6). In order to understand TCDD-induced

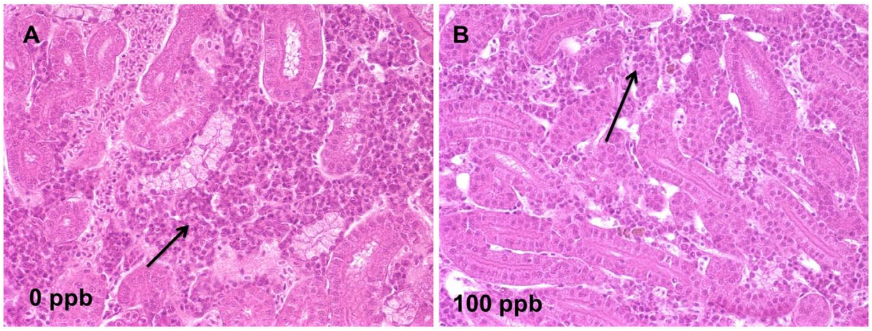

Figure 7. TCDD induced hemopoietic hypoplasia in kidney at 42 d. A) Normal hemopoietic tissue (arrow) in interstitium of anterior kidney of zebrafish in 0 ppb group; B) depletion of hemopoietic tissue (arrow) from interstitium of anterior kidney of zebrafish in 100 ppb group at 42 d (40X).
doi:10.1371/journal.pone.0100910.g007

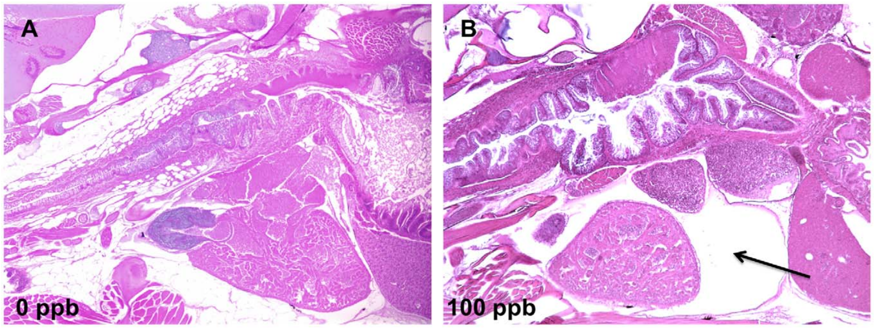

Figure 8. TCDD caused pericardial edema in zebrafish at 42 d. A) Normal heart in zebrafish of 0 ppb group; B) pericardial edema (arrow) in fish fed 100 ppb TCDD at 42 d (5X).
doi:10.1371/journal.pone.0100910.g008

toxicity in female fish, especially with regard to gonadal development, we compared the data of dysregulated genes between female and immature female fish using principal component analysis (PCA), which can evaluate differences among different samples based on dysregulated gene expression values. PCA showed a separation between mature and immature female fish using dysregulated genes from TCDD-treated female fish at each time point (Figure 11). Results from the PCA analysis (Figure 11) suggested that immature fish respond differently to TCDD than mature zebrafish. As a result, we could not consolidate female and immature fish into one group for gene expression analysis.

We compared IPA Canonical pathways from immature fish with female fish; and some significant pathways were identified only in immature fish are related to regulation of development and cell death, including apoptosis signaling, growth hormone signaling, TGF-β signaling (Table S11). Moreover, biomarkers for ovary development, such as vitellogenin 2 (*vtg2*), *vtg4 and vtg7*, were only found to be significantly down-regulated in immature fish.

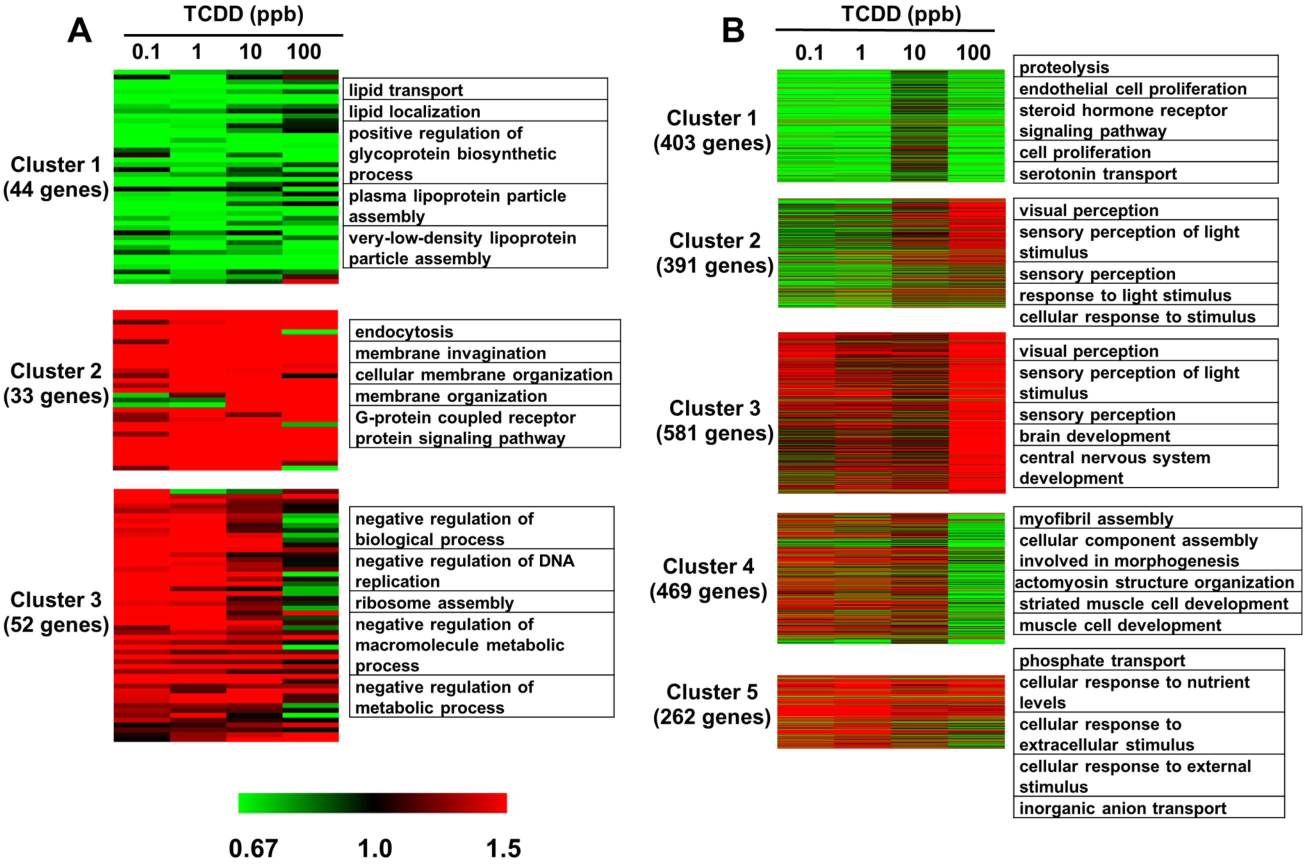

Figure 9. Cluster analysis (K-means) of the dysregulated genes with their representative Gene Ontology (GO) functional annotations from female (A) and male (B) zebrafish treated with dietary TCDD at 28 d (FDR ≤0.005). Data are mean fold changes for each treatment group. GO terms are referred to top five terms of Biological Process (BP) category.
doi:10.1371/journal.pone.0100910.g009

Table 2. Representative significant Canonical Pathways and Tox Lists from dysregulated genes in zebrafish treated with TCDD at 28 d.

Canonical pathways		Tox list	
**Female**	**Male**	**Female**	**Male**
Phototransduction Pathway	Calcium Signaling	Cytochrome P450 Panel - Substrate is a Sterol (Human)	Cardiac Hypertrophy
Lipid Antigen Presentation by CD1	Actin Cytoskeleton Signaling	Cytochrome P450 Panel - Substrate is a Sterol (Mouse)	Cytochrome P450 Panel - Substrate is a Vitamin (Mouse)
Virus Entry via Endocytic Pathways	Phototransduction Pathway	Cytochrome P450 Panel - Substrate is a Sterol (Rat)	Cytochrome P450 Panel - Substrate is a Vitamin (Rat)
IL-8 Signaling	Cellular Effects of Sildenafil (Viagra)	Aryl Hydrocarbon Receptor Signaling	Cytochrome P450 Panel - Substrate is a Sterol (Human)
CTLA4 Signaling in Cytotoxic T Lymphocytes	Protein Kinase A Signaling	Cell Cycle: G1/S Checkpoint Regulation	Cytochrome P450 Panel - Substrate is a Sterol (Mouse)
Pentose Phosphate Pathway	Tight Junction Signaling	p53 Signaling	Cytochrome P450 Panel - Substrate is a Sterol (Rat)
Cardiac Hypertrophy Signaling	Axonal Guidance Signaling	Renal Necrosis/Cell Death	PPARα/RXRα Activation
Role of RIG1-like Receptors in Antiviral Innate Immunity	ILK Signaling	NRF2-mediated Oxidative Stress Response	PXR/RXR Activation
GABA Receptor Signaling	Circadian Rhythm Signaling		Renal Proximal Tubule Toxicity Biomarker Panel (Rat)
Aryl Hydrocarbon Receptor Signaling	Ephrin Receptor Signaling		Cell Cycle: G2/M DNA Damage Checkpoint Regulation

doi:10.1371/journal.pone.0100910.t002

Expression of exon 4 within *vasa* was quantified by QPCR as shown in Figure 12 and Figure 13. In the dose-dependent experiment, expression of exon 4 was significantly up-regulated in male fish and significantly down-regulated in females (Figure 12). Expression of exon 4 in males and females treated with 100 ppb TCDD were significantly different from those in control males or females. In the time-dependent experiment, expression of exon 4 in males was up-regulated in both control females and immature female in the 100-ppb group (Figure 13); however, there was a sharp decrease in expression of exon 4 in 100 ppb TCDD-treated female fish at 28 d.

Discussion

Growth and accumulation of TCDD

Exposure of zebrafish embryos (2 h post-fertilization, hpf) to waterborne TCDD caused decreases in total body length and length of bone in the jaw of lava [24]. A study by Lanham *et al.* [25] showed that body weight of zebrafish was decreased by waterborne exposure to TCDD at 5 day post-fertilization (dpf) or 20–25 dpf, but decreased weight was not observed after 35 dpf, indicating that sensitivity of fish to TCDD exposure decreases during growth. In the present study, the juvenile fish were larger and older than those in the Lanham study; highest accumulated TCDD at 15.49 and 18.04 ppb in female and male fish did not cause significant mortality, except for decreases in body weight at 28 d (Table S1). This indicates that life-stage and body size account for much of the sensitivity to TCDD in zebrafish.

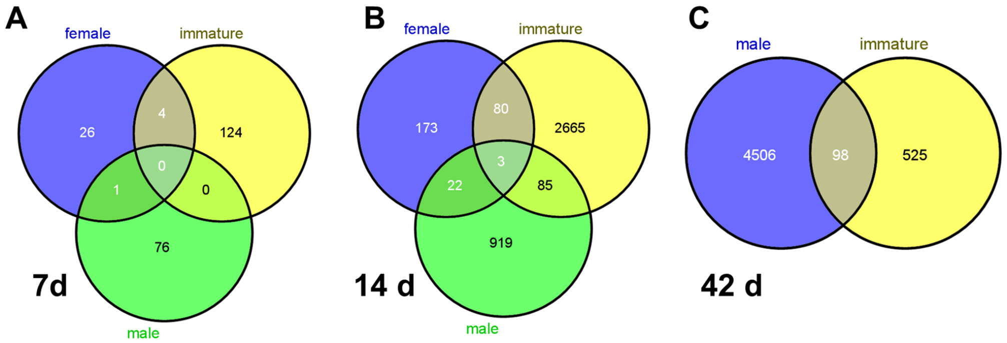

Figure 10. Venn diagram of dysregulated genes in female, immature female and male zebrafish treated with 100 ppb TCDD at 7 d (A), 14 d (B) and 42 d (C).
doi:10.1371/journal.pone.0100910.g010

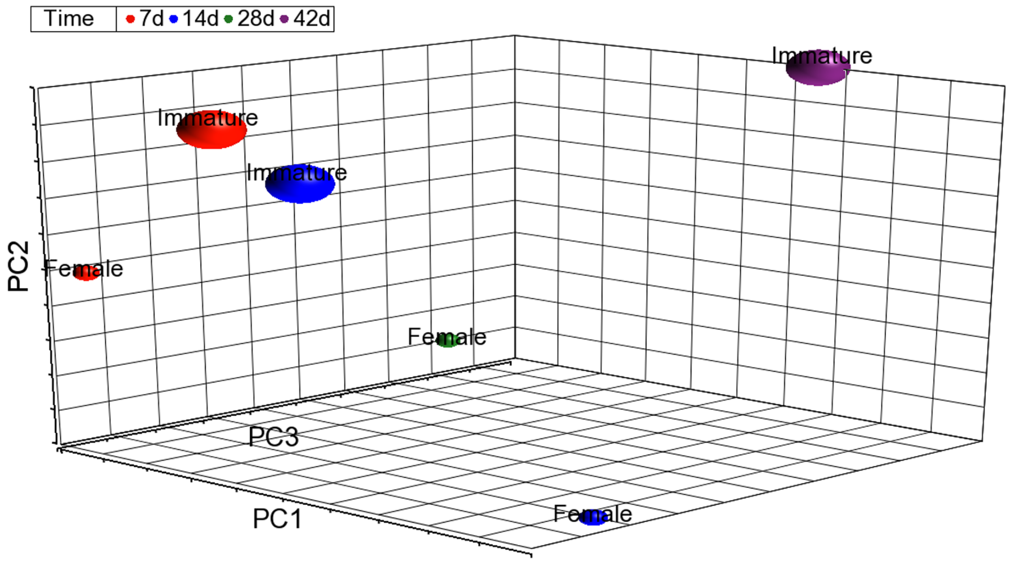

Figure 11. Principal component analysis (PCA) of dysregulated genes from TCDD-treated female fish at each time point. The gene list of 3679 genes for the principal component analysis was generated by combining all dysregulated genes from female and immature fish at each time point and removing duplicates. The value for each gene within each group was computed as the ratio of average of expression of treatment relative to the average of control expression levels for that gene.
doi:10.1371/journal.pone.0100910.g011

Additionally, differences between zebrafish and other fish species reflect the species-specific sensitivity to TCDD exposure [26,27], which might be due to the specific affinities of AhR to TCDD in different species [28]. Decreases in body length and weight in TCDD-treated zebrafish may be due to loss of nutrition because of TCDD-induced lesions in their digestive systems (oropharynx, liver, intestine and pancreas) found at 28 d and 42 d.

Previous study by King Heiden *et al.* [29] showed that dietary TCDD exposure (applied doses at 43 ppb) caused 36 ppb in whole-body fish at 20 d; and TCDD is highly accumulates in lipid-rich organs, such as ovary. In the present study, assimilations of TCDD in both females and males exhibited dose- and time-dependent increases in whole fish (Figure 3). Dietary TCDD at 100 ppb resulted in the assimilation of 15.49 and 18.04 ppb in female and male fish at 28 d, respectively, without significant mortality. Thus, the treatments we used for exposure in this project can be applied to sublethal pathologic/toxic research using zebrafish.

Lesions associated with dysregulation of gene expression in TCDD-treated zebrafish

Previous laboratory studies in mammals show that TCDD causes lesions in multiple organs, such as liver, lung [30] and dermis [31]. In teleost, pathologic alterations observed in zebrafish

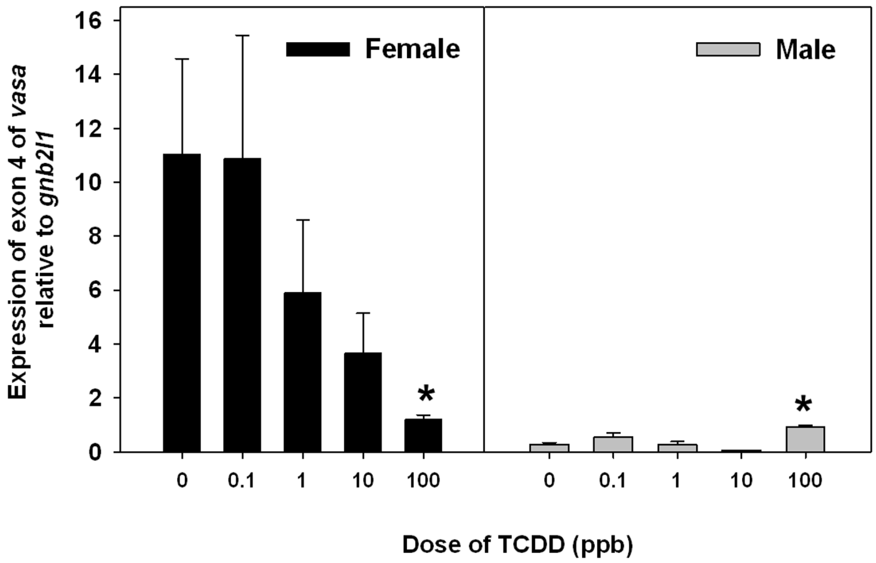

Figure 12. Expression of exon 4 of vasa in female and male zebrafish treated with TCDD at 28 d. The asterisk (*) means significant differences in exon 4 expression in 100 ppb group compared to control group with one-way ANOVA.
doi:10.1371/journal.pone.0100910.g012

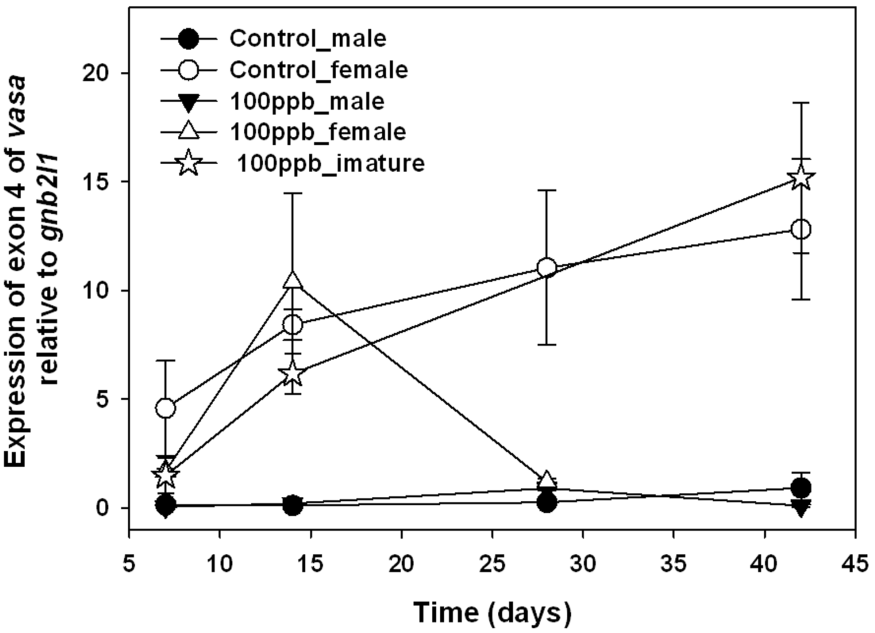

Figure 13. Expression of exon 4 of vasa in zebrafish treated with 100 ppb TCDD within 42 d. Immature female fish were found at 7, 14, and 42 d. And no fish in 100 ppb group were identified as female by physical observation at 42 d.
doi:10.1371/journal.pone.0100910.g013

were similar to those of other fish species. TCDD causes craniofacial malformations, disruption in jaw cartilage growth, yolk sac edema, altered heart rate, heat failure, and delayed development and death in embryos and larvae [7,29,32–35]. In adult fish, TCDD causes hepatic lipidosis and hypertrophy, glycogen depletion in liver, inhibition of fin regeneration and altered gonadogenesis [11,36,37]. In juvenile fish, the TCDD-induced lesions observed reflect similarity to exposed embryo/larvae and adults, including craniofacial malformations, degeneration in fin and gill, and altered pigmentation [25,38].

In the present study, lesions (Figure 4) identified in juvenile zebrafish showed certain similarities to previous research on juvenile yellow perch (*Perca flavescens*) treated with TCDD, such as decreases in hemopoietic cells in kidney marrow, depletion of adipose tissue, and edema syndromes [39]. Skeletal malformation and glycogen depletion in liver seen in the present study were also reported in larvae and adult fish [11,25,37]. However, altered pigmentation and fin necrosis were not observed, as was reported in 30 dpf juvenile zebrafish [25] and juvenile yellow perch [39]. TCDD was found to influence proliferation or differentiation processes of hemopoietic stem cells in bone marrow of mice after dietary exposure [40]. Unlike human beings, hemopoiesis occurs in kidney marrow of zebrafish instead of in bone marrow [41,42]. We found that the number of hemopoietic cells in kidney marrow was reduced in zebrafish treated with TCDD, and this may be correlated with the anemia observed in peripheral blood.

Results from IPA showed that ahr signaling was one of significant Canonical pathways and metabolism of xenobiotics by cytochrome P450 was one of significant Tox lists (Table 2). TCDD is known to bind ahr and arnt to mediate gene expression, such as cytochrome P450, family 1, subfamily A, cyp1a [43]. TCDD-induced oxidative stress can also trigger an apoptotic signal and induction of cytochrome P450 enzymes is a critical event in the apoptotic cascade [44]. Significant enrichment regarding NRF2-mediated oxidative stress response, p53 signaling, and cell cycle regulation indicated that the lesions induced by TCDD in fish might correlate with TCDD-induced oxidative stress and apoptosis. Tox Lists related to diseases of heart, kidney and liver were significantly represented in both the dose- and time-dependent experiments, including cardiac necrosis/cell death, cardiac fibrosis, long-term renal injury pro-oxidative response panel, renal necrosis/cell death, and liver necrosis/cell death (Tables S11 and S12). All of these results are highly correlated with chronic toxic effects of TCDD we observed in heart, kidney and liver (Figure 4).

TCDD-induced neuronal toxicities have mainly been reported in rats and mice following intraperitoneal injection of high doses. These include apoptosis in granule neuron cells, alterations in metabolism of neurotransmitters, formation of reactive oxygen species (ROS) in cerebellum, and dysfunction in motor, learning and visual abilities [45–49]. In zebrafish, embryonic exposure to TCDD reduces the number of neurons in the larval brain [50] and causes apoptosis in midbrain [51]. We reported the first evidence of TCDD-induced morphologic lesions in the olfactory organ of rainbow trout [38], in this study we found similar results from TCDD-treated zebrafish that exhibited highly disorganized and poorly differentiated hyperplastic lamellae (Figure 5). Evidence of TCDD-induced lesions in nasal epithelium of teleost is similar to results in rats which show hyperplastic and metaplastic changes within the nasal mucosa after chronic administration by gavage with TCDD [52].

Enriched GO terms in cluster 1, 2, 3 and 4 for treated male fish (Figure 9B) exhibited a correlation with nervous system development, such as endothelial cell proliferation (cluster 1), brain development and central nervous system development (cluster 3). Some enriched GO terms from male fish also related to eye development, such as sensory perception of light stimulus and overall visual perception. There is a single report of TCDD-induced decreases in the densitiy of retinal ganglion cells in rainbow trout [53] but nothing in zebrafish. We did not focus on eye development in this study, and potential effects of TCDD on eye development in zebrafish need to be further explored.

TCDD-induced disruption of ovarian development associated with gene expression in zebrafish

TCDD was found to inhibit the transition from the pre-vitellogenic stage to the vitellogenic stage in follicles of adult female zebrafish [36], and caused decreases in ovarian weight and ovarian necrosis [29]. Early-life waterborne exposure to TCDD caused impairment of reproductive capacity in both males and females as adults [7]. In the present study, we found that less than 30% of the collected fish contained a mature ovary when exposed to 10 ppb and none in the 100 ppb group at 42 d. This made it difficult for us to determine their sex by physical observation during sampling. Histopathologic analysis showed that ovaries were immature in fish treated with 100 ppb TCDD and that many contained immature cortical alveolar and perinucleolar oocytes, stages prior to the vitellogenic stage [54] (Figure 6). In addition, sex determination by PCR using *vasa* primers also confirmed that the fish with abnormal gonads were female (Figure 2 and Table 1). Our results are thereby similar to a previous study by Wannemacher *et al.* [55] that showed significant increases in the number of fish with immature, previtellogenic oocytes after dietary exposure to TCDD.

The *vasa* mRNA is expressed as two alternatively spliced transcripts (with or without exon 4) that are differentially expressed in males and females. The longer product that contains exon 4 is primarily expressed in females with very low expression in males [16]. Using our QPCR assay specifically targeting exon 4, we found that expression of exon 4 was down-regulated in females in both Dose- and Time-dependent experiments; however, expression of exon 4 in immature females is similar to that of normal female fish in the control group (Figures 12 and 13), indicating that TCDD might only influence splicing of *vasa* in female fish with matured ovaries. Previous studies use expression of *vasa* mRNA as a marker for primordial germ cells of zebrafish at early-life stages [56,57], however, little is to know about the function of *vasa* on sex-determination or ovarian development during juvenile stages.

We speculated that TCDD may inhibit the estrogen biosynthesis process by down-regulation of genes related to estrogen production, such as steroidogenic acute regulatory protein (*star*), *cyp11a1*, and *cyp19a1a* (aromatase), as we observed in our previous studies [12,36]. However, none of these genes was found to be dysregulated significantly by TCDD in immature fish except for *cyp17a1*, which showed a four-fold up-regulation at 14 d. We also evaluated genes related to regulation of ovarian development, such as follicle-stimulating hormone receptor (*fshr*), luteinizing hormone receptor (*lhcgr*), estrogen receptors (*esr1, esr2a, esr2b*), epidermal growth factor (*egf*) and egf receptor (*egfr*), and we found that only *esr1* and *efgr* were down-regulated at 7 d and 42 d in immature fish, respectively. Thus, our results cannot provide strong support for TCDD-induced disruption of ovarian development through inhibition of the estrogen biosynthesis pathway, but does suggest effects on receptor signaling involved in ovarian foliculogenesis. It is important to remember that, in this study, we performed microarray using RNA isolated from whole fish. In order to reveal transcriptional changes relevant to tissue-specific effects if may be necessary to use only mRNA from that tissue.

In the Great Lakes region, TCDD levels of 0.02–0.04 ppb have been reported in salmonid species (e.g., lake trout) [58,59]. We used much higher levels of TCDD in the laboratory since we wished to investigate the mechanisms of dietary TCDD toxicity in zebrafish, which are much more resistant to the toxic effects of TCDD than are salmonids using other means of exposure [26–28,60–61]. In a recently published parallel study using rainbow trout [38], we found that dietary TCDD caused overt toxicity in juvenile rainbow trout at wide range (from 0.1 ppb to 100 ppb). Zebrafish in this study were more resistant to dietary TCDD exposure as indicated by lower observed mortality and fewer pathologic alterations in the lower dose groups. In conclusion, chronic dietary TCDD exposure from juvenile stages delayed maturation of ovaries in female fish and caused lesions in multiple organs in zebrafish, and caused dose- and time-dependent TCDD accumulation. Functional and pathway analysis of microarray data using whole fish body revealed a correlation with various lesions and putative mechanisms for their genesis. As for the disruption of ovarian development and lesions in nasal epithelium induced by TCDD, tissue-specific microarrays should be performed to explore the mechanisms.

Supporting Information

Table S1 Body weight, length and condition factor (CF) of male and female zebrafish during 42 d of dietary TCDD exposure.
(XLSX)

Table S2 Epithelial lesions in various organs of zebrafish sampled after 28 d of dietary exposure to TCDD.
(DOCX)

Table S3 Epithelial lesions in various organs of zebrafish sampled after 42 d of dietary exposure to TCDD.
(DOCX)

Table S4 Mesenchymal lesions in various organs of zebrafish sampled after 28 d of dietary exposure to TCDD.
(DOCX)

Table S5 Mesenchymal lesions in various organs of zebrafish sampled after 42 d of dietary exposure to TCDD.
(DOCX)

Table S6 Edema syndrome lesions in zebrafish sampled after 28 d weeks of dietary exposure to TCDD.
(DOCX)

Table S7 Edema syndrome lesions in zebrafish fry sampled after 42 d of dietary exposure to TCDD.
(DOCX)

Table S8 Dysregulated genes in female zebrafish after 28 d of exposure to TCDD (FDR0.05).
(XLSX)

Table S9 Dysregulated genes in male zebrafish after 28 d of exposure to TCDD (FDR0.05).
(XLSX)

Table S10 Dysregulated genes in male, female and immature zebrafish at 7 d, 14 d, 28 d and 42 d of exposure to 100 ppb TCDD (FDR0.05).
(XLSX)

Table S11 Representative significant Canonical Pathways and Tox Lists from dysregulated genes in zebrafish treated with 100 ppb TCDD at 7, 14 or 42 d.
(XLSX)

Table S12 Representative significant Canonical Pathways and Tox Lists from dysregulated genes in zebrafish treated with TCDD at 28 d.
(XLSX)

Acknowledgments

Many thanks to Kendrick Wall, Susan Miller, Arie Brenner and Matt Pickens for their technical assistance. This paper has been submitted by

Qing Liu in partial fulfillment of the requirements for the Ph.D. degree in Biological Sciences at the University of Wisconsin-Milwaukee.

Author Contributions

Conceived and designed the experiments: MJC QL RJH. Performed the experiments: QL JMS RC. Analyzed the data: QL JMS RC. Contributed reagents/materials/analysis tools: JMS RC CYH NJ GG PJT MJC. Wrote the paper: QL MJC PJT RJH. Expertise and advice for transcriptomic data analysis: CYH NJ GG PJT.

References

1. White SS, Birnbaum LS (2009) An overview of the effects of dioxins and dioxin-like compounds on vertebrates, as documented in human and ecological epidemiology. J Environ Sci Health C Environ Carcinog Ecotoxicol Rev 27: 197–211.
2. Bertazzi PA, Bernucci I, Brambilla G, Consonni D, Pesatori AC (1998) The Seveso studies on early and long-term effects of dioxin exposure: a review. Environ Health Perspect 106: 625–633.
3. Humblet O, Birnbaum L, Rimm E, Mittleman MA, Hauser R (2008) Dioxins and cardiovascular disease mortality. Environ Health Perspect 116: 1443–1448.
4. Ivnitski I, Elmaoued R, Walker MK (2001) 2,3,7,8-tetrachlorodibenzo-p-dioxin (TCDD) inhibition of coronary development is preceded by a decrease in myocyte proliferation and an increase in cardiac apoptosis. Teratology 64: 201–212.
5. Steenland K, Piacitelli L, Deddens J, Fingerhut M, Chang LI (1999) Cancer, heart disease, and diabetes in workers exposed to 2,3,7,8-tetrachlorodibenzo-p-dioxin. J Natl Cancer Inst 91: 779–786.
6. Carvan MJ 3rd, Gallagher EP, Goksøyr A, Hahn ME, Larsson DG (2007) Fish models in toxicology. Zebrafish 4: 9–20.
7. King Heiden TC, Spitsbergen J, Heideman W, Peterson RE (2009) Persistent adverse effects on health and reproduction caused by exposure of zebrafish to 2,3,7,8-tetrachlorodibenzo-p-dioxin during early development and gonad differentiation. Toxicol Sci 109: 75–81.
8. Yamauchi M, Kim EY, Iwata H, Shima Y, Tanabe S (2006) Toxic effects of 2,3,7,8-tetrachlorodibenzo-p-dioxin (TCDD) in developing red seabream (Pagrus major) embryo: an association of morphological deformities with AHR1, AHR2 and CYP1A expressions. Aquat Toxicol 80: 166–179.
9. Bello SM, Heideman W, Peterson RE (2004) 2,3,7,8-Tetrachlorodibenzo-p-dioxin inhibits regression of the common cardinal vein in developing zebrafish. 2,3,7,8-Tetrachlorodibenzo-p-dioxin inhibits regression of the common cardinal vein in developing zebrafish. Toxicol Sci 78: 258–266.
10. Teraoka H, Kubota A, Dong W, Kawai Y, Yamazaki K, et al. (2009) Role of the cyclooxygenase 2-thromboxane pathway in 2,3,7,8-tetrachlorodibenzo-p-dioxin-induced decrease in mesencephalic vein blood flow in the zebrafish embryo. Toxicol Appl Pharmacol 234: 33–40.
11. Zodrow JM, Tanguay RL (2003) 2,3,7,8-tetrachlorodibenzo-p-dioxin inhibits zebrafish caudal fin regeneration. Toxicol Sci 76: 151–161.
12. King Heiden TC, Struble CA, Rise ML, Hessner MJ, Hutz RJ, et al. (2008) Molecular targets of 2,3,7,8-tetrachlorodibenzo-p-dioxin (TCDD) within the zebrafish ovary: insights into TCDD-induced endocrine disruption and reproductive toxicity. Reprod Toxicol 25: 47–57.
13. Antignac JP, Marchand P, Gade C, Matayron G, Qannari EM, et al. (2006) Studying variations in the PCDD/PCDF profile across various food products using multivariate statistical analysis. Anal Bioanal Chem 384: 271–279.
14. Laurent C, Marchand P, Feidt C, Le Bizec B, Rychen G (2005) Tissue distribution and bioconcentration factors of PCDD/F in the liver and adipose tissue following chronic ingestion of contaminated milk in rats. Chemosphere 60: 929–938.
15. Marchand P, Cariou R, Vénisseau A, Brosseaud A, Antignac JP, et al. (2010) Predicting PCDD/F and dioxin-like PCB contamination levels in bovine edible tissues from in vivo sampling. Chemosphere 80: 634–640.
16. Krøvel AV, Olsen LC (2004) Sexual dimorphic expression pattern of a splice variant of zebrafish vasa during gonadal development. Dev Biol 271: 190–197.
17. Pfaffl MW (2001) A new mathematical model for relative quantification in real-time RT-PCR. Nucleic Acids Res 29: e45.
18. Irizarry RA, Hobbs B, Collin F, Beazer-Barclay YD, Antonellis KJ, et al. (2003) Exploration, normalization, and summaries of high density oligonucleotide array probe level data. Biostatistics 4: 249–264.
19. Saeed AI, Sharov V, White J, Li J, Liang W, et al. (2003) TM4: a free, open-source system for microarray data management and analysis. Biotechniques 34: 374–378.
20. Saeed AI, Bhagabati NK, Braisted JC, Liang W, Sharov V, et al. (2006) TM4 microarray software suite. Methods Enzymol 411: 134–193.
21. Benjamini Y, Hochberg Y (1995) Controlling the false discovery rate: a practical and powerful approach to multiple testing. Journal of the Royal Statistical Society Series B 57: 289–300.
22. Carbon S, Ireland A, Mungall CJ, Shu S, Marshall B, et al. (2009) AmiGO: online access to ontology and annotation data. Bioinformatics 25: 288–289.
23. Lonergan KM, Chari R, Coe BP, Wilson IM, Tsao MS, et al. (2010) Transcriptome profiles of carcinoma-in-situ and invasive non-small cell lung cancer as revealed by SAGE. PLoS One 5: e9162.
24. Hill A, Howard V, Cossins A (2004) Characterization of TCDD-induced craniofacial malformations and retardation of zebrafish growth. Journal of Fish Biology 64: 911–922.
25. Lanham KA, Peterson RE, Heideman W (2012) Sensitivity to dioxin decreases as zebrafish mature. Toxicol Sci 127: 360–370.
26. Elonen GE, Spehar RL, Holcombe GW, Johnson RD, Fernandez JD, et al. (1998) Comparative toxicity of 2,3,7,8-tetrachlorodibenzo-p-dioxin to seven freshwater fish species during early life-stage development. Environ Toxicol Chem 17: 472–483.
27. Kleeman JM, Olson JR, Peterson RE (1988) Species differences in 2,3,7,8-tetrachlorodibenzo-p-dioxin toxicity and biotransformation in fish. Fundam Appl Toxicol 10: 206–213.
28. Abnet CC, Tanguay RL, Heideman W, Peterson RE (1999) Transactivation activity of human, zebrafish, and rainbow trout aryl hydrocarbon receptors expressed in COS-7 cells: greater insight into species differences in toxic potency of polychlorinated dibenzo-p-dioxin, dibenzofuran and biphenyl congeners. Toxicol Appl Pharmacol 159: 41–51.
29. King Heiden TC, Hutz RJ, Carvan MJ 3rd (2005) Accumulation, tissue distribution, and maternal transfer of dietary 2,3,7,8,-tetrachlorodibenzo-p-dioxin: impacts on reproductive success of zebrafish. Toxicol Sci 87: 497–507.
30. Kociba RJ, Keyes DG, Beyer JE, Carreon RM, Wade CE, et al. (1978) Results of a two-year chronic toxicity and oncogenicity study of 2,3,7,8-tetrachlorodibenzo-p-dioxin in rats. Toxicol Appl Pharmacol 46: 279–303.
31. Ito T, Inouye K, Nohara K, Tohyama C, Fujimaki H (2008) TCDD exposure exacerbates atopic dermatitis-related inflammation in NC/Nga mice. Toxicol Lett 177: 31–37.
32. DS Antkiewicz, CG Burns, SA Carney, RE Peterson, W Heideman (2005) Heart malformation is an early response to TCDD in embryonic zebrafish. Toxicol Sci 84: 368–377.
33. Hornung MW, Spitsbergen JM, Peterson RE (1999) 2,3,7,8-Tetrachlorodibenzo-p-dioxin alters cardiovascular and craniofacial development and function in sac fry of rainbow trout (Oncorhynchus mykiss). Toxicol Sci 47: 40–51.
34. Spitsbergen JM, Walker AK, Olson JR, Peterson RE (1991) Pathologic alterations in early life stages of lake trout, Salvelinus namaycush, exposed to 2,3,7,8-tetrachlorodibenzo- p-dioxin as fertilized eggs. Aquatic Toxicology 19: 41–71.
35. Teraoka H, Dong W, Ogawa S, Tsukiyama S, Okuhara Y, et al. (2002) 2,3,7,8-Tetrachlorodibenzo-p-dioxin toxicity in the zebrafish embryo: altered regional blood flow and impaired lower jaw development. Toxicol Sci 65: 192–199.
36. King Heiden TC, Carvan MJ 3rd, Hutz RJ (2006) Inhibition of follicular development, vitellogenesis, and serum 17beta-estradiol concentrations in zebrafish following chronic, sublethal dietary exposure to 2,3,7,8-tetrachlorodibenzo-p-dioxin. Toxicol Sci 90: 490–499.
37. Zodrow JM, Stegeman JJ, Tanguay RL (2004) Histological analysis of acute toxicity of 2,3,7,8-tetrachlorodibenzo-p-dioxin (TCDD) in zebrafish. Aquat Toxicol 66: 25–38.
38. Liu Q, Rise ML, Spitsbergen JM, Hori TS, Mieritz M, et al. (2013) Gene expression and pathologic alterations in juvenile rainbow trout due to chronic dietary TCDD exposure. Aquat Toxicol 140–141: 356–368.
39. Spitsbergen JM, Kleeman JM, Peterson RE (1988) 2,3,7,8-Tetrachlorodibenzo-p-dioxin toxicity in yellow perch (Perca flavescens). J Toxicol Environ Health 23: 359–383.
40. Murante FG, Gasiewicz TA (2000) Hemopoietic progenitor cells are sensitive targets of 2,3,7,8-tetrachlorodibenzo-p-dioxin in C57BL/6J mice. Toxicol Sci 54: 374–383.
41. Amatruda JF, Zon LI (1999) Dissecting hematopoiesis and disease using the zebrafish. Dev Biol 216: 1–15.
42. Ivanovski O, Kulkeaw K, Nakagawa M, Sasaki T, Mizuochi Ch, et al. (2009) Characterization of kidney marrow in zebrafish (Danio rerio) by using a new surgical technique. Prilozi 30: 71–80.
43. Hutz RJ, Carvan MJ 3rd, Baldridge MG, Conley LK, Heiden TK (2006) Environmental toxicants and effects on female reproductive function. Tren Reprod Bio 2: 1–11.
44. Nebert DW, Roe AL, Dieter MZ, Solis WA, Yang Y, et al. (2000) Role of the aromatic hydrocarbon receptor and [Ah] gene battery in the oxidative stress response, cell cycle control, and apoptosis. Biochem Pharmacol 59: 65–85.
45. Unkila M, Pohjanvirta R, Tuomisto J (1995) Biochemical effects of 2,3,7,8-tetrachlorodibenzo-p-dioxin (TCDD) and related compounds on the central nervous system. Int J Biochem Cell Biol 27: 443–455.
46. Seo BW, Sparks AJ, Medora K, Amin S, Schantz SL (1999) Learning and memory in rats gestationally and lactationally exposed to 2,3,7,8-tetrachlorodibenzo-p-dioxin (TCDD). Neurotoxicol Teratol 21: 231–239.
47. Markowski VP, Zareba G, Stern S, Cox C, Weiss B (2001) Altered operant responding for motor reinforcement and the determination of benchmark doses following perinatal exposure to low-level 2,3,7,8-tetrachlorodibenzo-p-dioxin. Environ Health Perspect 109: 621–627.

48. Kim SY, Yang JH (2005) Neurotoxic effects of 2,3,7,8-tetrachlorodibenzo-p-dioxin in cerebellar granule cells. Exp Mol Med 37: 58–64.
49. Collins LL, Williamson MA, Thompson BD, Dever DP, Gasiewicz TA, et al. (2008) 2,3,7,8-Tetracholorodibenzo-p-dioxin exposure disrupts granule neuron precursor maturation in the developing mouse cerebellum. Toxicol Sci 103: 125–136.
50. Hill A, Howard CV, Strahle U, Cossins A (2003) Neurodevelopmental defects in zebrafish (Danio rerio) at environmentally relevant dioxin (TCDD) concentrations. Toxicol Sci 76: 392–399.
51. Dong W, Teraoka H, Yamazaki K, Tsukiyama S, Imani S, et al. (2002) 2,3,7,8-tetrachlorodibenzo-p-dioxin toxicity in the zebrafish embryo: local circulation failure in the dorsal midbrain is associated with increased apoptosis. Toxicol Sci 69: 191–201.
52. Nyska A, Yoshizawa K, Jokinen MP, Brix AE, Sells DM, et al. (2005) Olfactory epithelial metaplasia and hyperplasia in female Harlan Sprague-Dawley rats following chronic treatment with polychlorinated biphenyls. Toxicol Pathol 33: 371–377.
53. Carvalho PS, Tillitt DE (2004) 2,3,7,8-TCDD effects on visual structure and function in swim-up rainbow trout.\. Environ Sci Technol 38: 6300–6306.
54. West G (1990) Methods of Assessing Ovarian development in Fishes: a Review. Australian Journal of Marine and Freshwater Research 41: 199–222.
55. Wannemacher R, Rebstock A, Kulzer E, Schrenk D, Bock KW (1992) Effects of 2,3,7,8-tetrachlorodibenzo-p-dioxin on reproduction and oogenesis in zebrafish (Brachydanio rerio). Chemosphere 24: 1361–1368.
56. Braat AK, Zandbergen T, van de Water S, Goos HJ, Zivkovic D (1999) Characterization of zebrafish primordial germ cells: morphology and early distribution of vasa RNA. Dev Dyn 216: 153–167.
57. Knaut H, Pelegri F, Bohmann K, Schwarz H, Nüsslein-Volhard C (2000) Zebrafish vasa RNA but not its protein is a component of the germ plasm and segregates asymmetrically before germline specification. J Cell Biol 149: 875–888.
58. Ryan JJ, Lau PY, Pilon JC, Lewis D, McLeod HA, et al. (1984) Incidence and levels of 2,3,7,8-tetrachlorodibenzo-p-dioxin in Lake Ontario commercial fish Environ. Sci. Technol.18: 719–721.
59. Turyk ME, Bhavsar SP, Bowerman W, Boysen E, Clark M, et al. (2012) Environ Health Perspect.120(1): 11–8.
60. Hill AJ, Teraoka H, Heideman W, Peterson RE (2005) Zebrafish as a model vertebrate for investigating chemical toxicity. Toxicol Sci. 86(1): 6–19.
61. Carvan MJ 3rd, King Heiden TC, Tomasiewicz H (2005) The utility of zebrafish as a model for toxicological research. In: Mommsen TP and Moon TW, editors. Biochemistry and Molecular Biology of Fishes. Vol.6: 3–41.
